# Variance in multiplex suspension array assays: intraplex method improves reliability

**DOI:** 10.1186/1742-4682-4-32

**Published:** 2007-08-29

**Authors:** Brian Hanley

**Affiliations:** 1 Microbiology Graduate Group, University of California, Davis, CA 95616, USA; 2 BW Education and Forensics, 2710 Thomes Avenue, Cheyenne, Wyoming 82001, USA

## Abstract

**Background:**

Flow cytometry based suspended microarray assays are susceptible to many sources of variance; multi-well replication and inter-instrument reproducibility is uncertain.

**Method and results:**

An "intraplex" method was developed in order to minimize differences in sample readings between instruments. A full intraplex assay consists of a set *m *of microparticle set classifications assaying for the same analyte, with each of the *m *classifier sets having different sensitivity to analyte, and *n *classifier sets replicating each of the *m *levels of sensitivity, where *m *> 1 (generally *m *> 4 would be used).

**Conclusion:**

The intraplex method can compensate adequately for the sources of variance that have been identified in suspended microarray assays. It requires no changes to current equipment in use, and is a superior method of constructing precision assays. Additionally, Luminex^® ^users may want to consider the evidence that shows that despite calibration to the same standard, two instruments may not give similar results for all concentrations of analytes.

## Background

A suspended microarray assay system uses small particles, such as microspheres or microrods that contain some method for identifying a set of particles composing one assay. An chemical compound used to bind to a biological (or chemical) target molecule (analyte) is bound to the surface of a set of identical particles, which are generally in the size range of 3–15 microns. Differently labeled particles have different target molecules that they assay for. These particles are added to a liquid (such as serum or cell lysate) containing the potential analytes. (In systems such as "smart dust", the assay may be distributed in the field to detect analytes. A system such as "smart dust" may also use an alternative method of analyte signaling and readout.) The final step in the assay activates a reporter fluorophore that provides a signal. (Essentially, this is an ELISA assay on the surface of a small particle.) The particles are run through a flow cytometer, which may be optimized for the specific assay system. For each particle in the mixture, the cytometer identifies the classifier for the set the particle belongs to together with the fluorescence reading of the reporter fluorophore. Because the particle classifiers are designed to be unique for each analyte, it is possible to multiplex the assays together in a test tube in order to test for multiple analytes in one sample. Multi-well assay plates can be used to test many samples, and such assays then become a high throughput system.

The Luminex Corporation (Austin, Texas) is one vendor of specialized flow cytometry equipment, which they also license to BioRad (Bio-Rad Laboratories, Hercules, California). The Luminex assay examined in this study utilizes microspheres on the order of 5.6 microns in diameter, upon which antigens or antibodies have been covalently bonded (xMap™ assays). The Luminex xMap™ assay microspheres used in this study contain two classifier fluorophores. Each fluorophore has *n *levels of brightness that can be differentiated, and the two are proportionally varied to separate them into *n*^2 ^different microsphere populations for identification (currently *n*^2 ^= 100 for two fluorophores.) This study used classical sandwich assays to attach reporter molecules of streptavidin-linked phycoeryrthrin to the microspheres. Luminex also provides assays which utilize nucleotide hybridizations to attach reporter fluorophores, and other assays are possible. The reporter fluorophore intensity is then measured in a specialized flow cytometer together with the microsphere classifiers; the reporter fluorescence measurement is collected separately for each microsphere population in the mixture. For each microsphere classifier population a sample of microspheres is collected, and one or more of the following are then used as the reported value: median, mean, trimmed mean, or peak. Median is the most commonly used value. The system is usually deployed with one well containing the same analyte fluid, sometimes two, however, some laboratories use three replicate wells as a standard, and throw out outlier values when they occur.

The experimental sample fluid with *n *sets of microspheres flows up through a probe, which has a tip with 5 fine holes leading to a single channel at the top. The fluid travels through a system of tubing and valves into the flow cell, where (in the current equipment) two lasers are present. One laser stimulates the two marker fluorophores, and the other stimulates the reporter fluorophore. A system of avalanche photodiodes and a photomultiplier tube captures the fluorescence from marker and reporter emissions.

Users of the Luminex instrument with xMap™ microsphere arrays have had mixed success in correlating the results of the assays with ELISA assays and generating reproducible results for a given assay [1-7]. A solution offered at the Planet xMAP 2006 Symposium, where the results of a primarily Luminex authored paper [8] were presented, was to use more microspheres for each analyte. However, there are at least two significant matters not addressed by that recommendation: carryover between wells, and stochastic variance.

In response to the above, and a set of concerns from prior experimental work, the *intraplex *method was developed. This method compensates for various sources of variance that occur under typical real world laboratory conditions. Potential sources of variance that can be compensated for in whole or in part include: variation in size of microspheres affecting brightness [9]; carryover of microspheres between wells[10]; stochastic variance issues (unpublished); and inter-instrument calibration differences (response curve for varying concentrations of analyte by the complete opto-electronic system).

### Intraplex concept

In order to try to minimize inter-instrument and inter-well variances, the *intraplex *assay method was developed. Due to significant opportunities for confusion in this discussion, three terms are introduced for clarity: Suspended Microarray Particle (SMP), Suspended Microarray Particle Classifier Set (SMPCS) and Suspended Microarray Particle Classifier Set – IDentical Group (SMPCS-IDG). An SMP corresponds to a single microsphere, and an SMPCS corresponds to a set of microspheres that share a classifier. An SMPCS corresponds to what Luminex commonly calls "a microbead region", a "microbead set" or more colloquially, "a microbead" or simply "beads" and is usually interchangeable with bead number, since Luminex identifies their microbeads to users by numbers from 001 to 100 in the older systems in use.

What is new to intraplexing is the SMPCS-IDG, a superset of SMPCS's composing an identically responsive group. An SMPCS-IDG is a set of *n *SMPCS's that assay for the same analyte with the same level of sensitivity. This is explained in more detail below.

**Figure 1:**
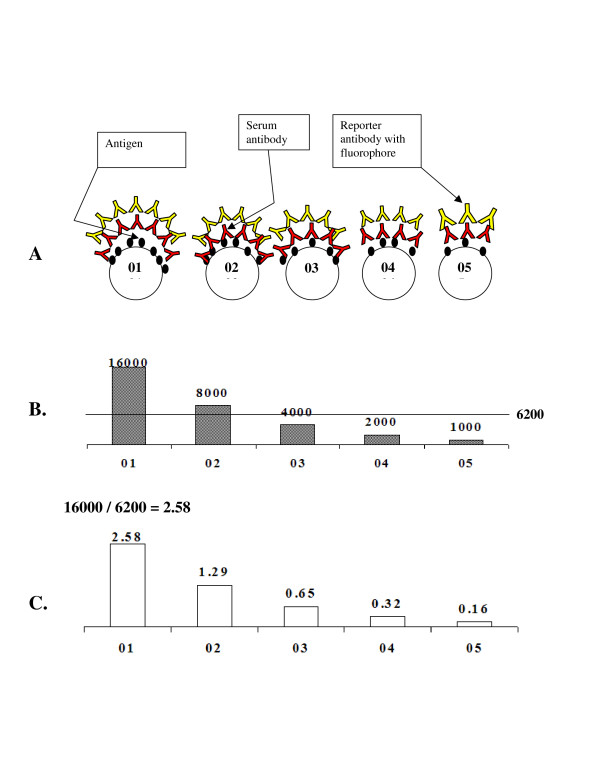
Simple intraplex concept diagram showing idealized characteristics. A. *m *= 5 different microsphere sets (i.e. 5 SMPCS's) (labeled 01 to 05) are shown. Their respective coatings of ligand (in this case antigen) to bind analyte (in this case antibody) are varied by consecutive dilutions. So, more binding sites are available for a target antibody analyte on those microspheres incubated with higher concentrations. B. Reporter fluorescence readings for an assay that reflect the 2× series dilutions of *ligand bound to microspheres *showing how each set responds differently to *the same *concentration of analyte. Mean of *m *= 6200 as denoted by horizontal line. This is the internal self mean of the *m *fluorescence readings. C. Internal self mean ratios for each of the SMPCS's. Example calculation shown for SMPCS 01. The mean of *m *is used as the denominator for each of the *m *fluorescence readings.

The *simple intraplex *shown in Figure 1 consists of *m *SMPCS's, all of which assay for the same analyte, but at differing levels of sensitivity. Having differing sensitivity to analyte results in different levels of signal from the reporter (typically a fluorophore) for each SMPCS. Figure 1 conceptualizes an antigen-on-microsphere type of assay, but the assay can be of any type. In this diagram, SMPCS's were made titrating to generate differing fluorescent intensities. This diagram is idealized, with each reading precisely half the one preceding it. In practice, SMPCS's will not differ so precisely. Figure 1 (C) illustrates one type of ratio, the ratio of each of the fluorescent reporter readings to the internal self-mean, which was found to be the most stable for generating replicated well assay readings. The internal self mean is produced by averaging *m *reporter readings to produce the mean of *m*. The mean of *m *is used as the denominator for each of the *m *readings. The end result is *m *internal self-mean ratios of the fluorescent readings of *m *to the mean of *m*. These ratios have been shown to be stable between instruments and between wells, even when absolute readings differ from each other by ratios as large as 30:1. It should be emphasized, however, that intraplexing cannot compensate for errors generated on the bench or in sample handling.

**Figure 2:**
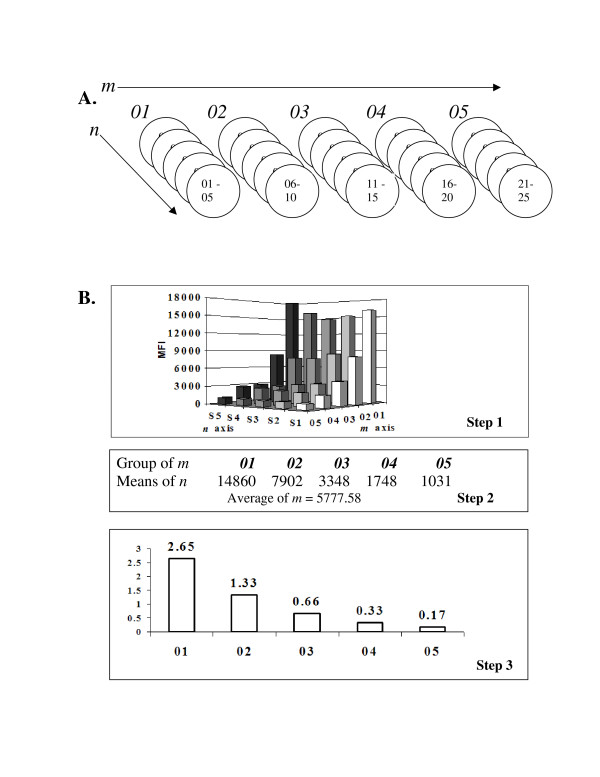
Concept diagram for m × n microsphere matrix. A. Each circle in this diagram represents a set of microspheres (i.e. an SMPCS). Each of the superset identical groups (i.e. SMPCS-IDG) (*m *= 5) of are coated at different sensitivities. The SMPCS-IDG's of *m *are across the top, labeled 01–05. Note that now each *m *is a superset composed of 5 microsphere set identifiers (i.e. an SMPCS-IDG). Each of *n *(01 to 05 for SMPCS-IDG 01, 06 to 10 for SMPCS-IDG 02, ...) microspheres that make up the superset SMPCS-IDG for *m *is coated in the same batch for identical sensitivity. Like figure 1, the *m *SMPCS-IDG's have serial dilutions (or some other useful difference in sensitivity method) in their manufacture. B. Processing of the intraplex using a simulated example. Step 1: On left is an *m *= 5 × *n *= 5 fluorescent reporter reading dataset graph for all SMPCS's, 01–25. (Note the outlier at 05, S5 that was removed for the set of *n *for the *m *SMPCS-IDG number 01.) Completion of step 1 is removal of outliers. Step 2: The result of this step is *m *averages, (means of *n*) using as input the *n *microsphere set fluorescence readings for each SMPCS-IDG. This is shown in the table. Each of these 5 means of *n *are averaged together to give a single mean of *m*. Step 3: Internal self mean ratios using the mean of *m *as denominator for each of the means of *n *from step 2. This is done in the same way as for the simple intraplex of figure 1.

The *full intraplex *conceptualized in Figure 2 is composed of an *m *× *n *matrix in which each of *m *different SMPCS-IDG's has *n *SMPCS's designed to be identical. This allows three levels of processing to be conducted on the readings. For example, analyzing the concentration of a single analyte, an *m *= 5 × *n *= 5 matrix could be developed. It would contain 5 SMPCS-IDG's, each containing 5 SMPCS's. Production of each of the 5 SMPCS-IDG's would usually be done together in a single batch, guaranteeing that all microspheres in each set should have the same average signal response level.

When processing this *5 *× *5 *intraplex, the first step of processing removes outlier values from each of the *5 *SMPCSs making up each SMPCS-IDG if outliers exist. Step two averages the remaining *n *readings for each of the *5 *sets, to obtain *5 *averages, or "means of *n*." Then these means of *n *are themselves averaged to produce a single mean of *m*. The third step uses the mean of *m *as the denominator for each of the 5 means of *n*. (i.e. essentially the same as for the simple intraplex above, with greater statistical confidence generated for each of the *m *SMPCS-IDG's.) Like the *simple intraplex*, the end result is 5 ratios, called internal self-mean ratios. This complete technique should give a high level of precision where precision is needed.

## Methods

### Preparation of xMap™ microspheres

Microsphere preparation was done according to standard Luminex xMap™ microsphere coating protocols. The assays used had already been tested against rhesus serum samples and levels of signal were recorded. This signal level was accepted as sufficient indication that they were representative of a real world assay.

The virus antigens used in these experiments were:

CMV- Cytomegalovirus,

SFV- Simian Foamy Virus,

SRV- Simian Type D Retrovirus,

SIV- Simian Immunodeficiency Virus.

The Luminex microsphere classifiers used for the four antigens are listed in Table 1. A 100s digit was prefixed to differentiate in-house assays from those acquired from outside (106 = microsphere region 006, 112 = microsphere region 012, etc.)

**Table 1:** Assays and microsphere classifiers available for use

**CMV**	**SFV**	**SRV**	**SIV**
106	105	146	104
112	111	147	133
113	115	152	137
180	118	197	
	166	198	
	173		

### Preparation of microtiter plates

MultiScreen HTS, BV (Millipore; Bedford, MA) 96 well filter plates were utilized for all assays. Preliminary studies of pipetting error indicated that volumes above 5 μl would have minimal error. All assays were conducted such that no fluid volume below 5 μl would be pipetted, and pipetting was done using a multi-channel pipetter. On the basis of preliminary evaporation studies, a total volume of at least 90 μl per well was used during incubations to minimize evaporation as a source of variance. In addition, all wells were filled within 2 minutes or less after each washing so that any difference between well concentrations due to evaporation was further minimized.

### Experiments

Using a setting to collect a minimum of 100 microspheres per sample, 3, 4, and 5 microsphere set intraplexes were used to assay for the same analyte. Serum titrations of 1:50, 1:100 and 1:200 were used with 32 replicate wells per titration. The aim was to find a method for improving the accuracy of xMap™ assays through better intra-well controls. In total, 25 SMPCS's (i.e. xMap™ microsphere regions) were multiplexed, including all elements of the intraplexes. One SMPCS was coated with BSA as a control to measure nonspecific binding. An additional set of 6 uncoated SMPCS's were used as an alternate experimental intraplex control.

The assays used in this study were developed previously for a simian virus detection project. They were manufactured using carboxylate xMap™ microspheres from Luminex (Luminex; Austin, TX) conjugated to multiple viral antigens; the viral antigens used were 4 microsphere sets for CMV, 5 sets for SFV, 5 for SRV and 3 for SIV. (Table 1) These assays, intended to bind Rhesus macaque antibody, were antigen attached to microspheres. The single Rhesus macaque serum used is known positive for SRV, CMV and SFV. This serum is known to be negative for SIV.

Three controls were used: uncoated microspheres, the SIV microsphere assays, and a BSA standard control for background. Serum from a single Rhesus macaque with known positive and negative characteristics for the assays used was the sole experimental sample (and thus a type of control). Samples were incubated for two hours on a shaker table, washed with PBS-Tween, then incubated for 40 minutes with R-Phycoerythrin-conjugated Affinipure F(ab) Fragment Goat anti-Human IgG Fcγ (Jackson ImmunoResearch Laboratories, Inc.; West Grove, PA), which was used as a conjugate reporter to detect the Rhesus macaque antigen specific IgG antibodies bound to antigen on microspheres. The plate contents were then washed with PBS-Tween, shaken to suspend the microspheres, washed again, resuspended, then read on a Luminex instrument. Plates were stored overnight at 4°C in a refrigerator and read on a Bioplex instrument the following morning.

### Data collection

Two instruments were used for these experiments: a Luminex Model 100 that is approximately 5 years old, and a Biorad Bioplex instrument installed in late December 2005 and commissioned for use in January 2006. Both instruments were under standard service contract. Prior to commencing the study, both instruments had been serviced by field technicians within the previous 2 months. Also prior to commencing the study, the Luminex instrument was upgraded to the latest software and firmware levels.

Each plate was run on both machines, first on the Luminex, and second on the Biorad Bioplex. Seven different statistics available from Luminex and Bioplex instrument software were examined for each instrument: mean, standard deviation, trimmed mean, median, trimmed standard deviation, peak and trimmed peak.

The mean is the simple arithmetic average of all fluorescent intensities for the microsphere set that pass gating criteria. The standard deviation is the standard deviation of the simple mean calculation. The trimmed mean is an average of the fluorescent intensities collected in a sample, using an algorithm that appears to remove data points on both sides of the median. The trimmed standard deviation is the standard deviation of the data points used in calculating the trimmed mean. The median is the most commonly used value for most instrument users.

Peak and trimmed peak values were not used because the Bioplex XML file does not present the "peak" values that are present in the Luminex CSV file. The peak value should correspond to a mode. Examination of distributions of individual microsphere events was done using data from the Bioplex XML file. However, these showed enough complexity, and since the precise algorithm used by the Luminex was unknown, attempting to calculate a facsimile peak value from Bioplex XML data was abandoned. Thus, it was not possible to include these data as a further test of normality of distribution for both datasets.

Distributions were examined for normality, focusing on what is usually available to users of the instrument. A simple preliminary test for normality of the distribution is to divide the mean by the median and the peak (mode) for the datasets. If the sample distribution is normal then these values are equal and the ratio is 1:1. If it is skewed, then the mean will be some multiple of the median if the skew is toward the high end, or some fraction of the median if the skew is towards the low end. While this test would not be correct under all conditions in the absence of the peak values, visual examination of some histograms of microsphere distributions taken from the Bioplex shows that it appears adequate for this instrument.

**Figure 3:**
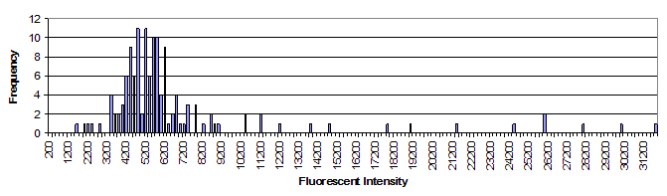
Histogram of intensities of reporter fluorophore for microsphere classifier set #97, that has an *N *of 136. This is a representative sample of the histograms generated by extracting event data from the Bio-Rad Bioplex XML data file. Visual inspection shows a fairly normal distribution with high end outliers in a long tail.

The fluorescent intensity histogram can be examined for each microsphere set, and the skew and normality could be determined directly. However, this information is only available from the Bio-Rad instrument in the XML export file. This study generated histograms for a significant number of wells, examined them, and determined that they approximated normal distributions, as exampled in Figure 3.

**Figure 4:**
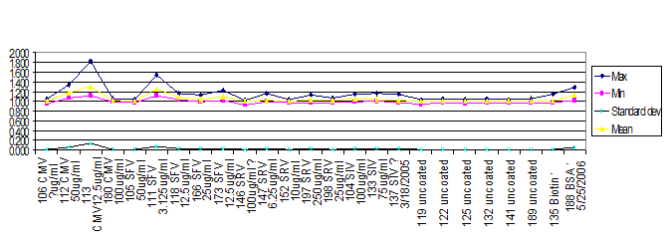
Ratio of trimmed mean/median. For this study, it was useful to use trimmed mean so that standard deviations would be available for each reading. This graph shows that the trimmed mean is close to the median which is commonly used for this instrument. This is also a strong indication of normal distribution. Y axis is mean fluorescent intensity (MFI).

Generally speaking, untrimmed mean data for a microsphere set can be skewed (Figure 3). It was consistent that skews seen were mostly to the high side owing to a small number of outliers. The instrument output contains a trimmed mean value. Trimmed mean/median ratios on a well by well basis gave ratios close to 1:1 (Figure 4). Thus, it makes sense that Figure 4 shows a small amount of residual high side skew for trimmed means in some cases.

This examination showed that trimmed mean and trimmed standard deviation was the optimum data source for the instrument for this study, since analysis used standard deviations of individual readings (not shown), although the median is more commonly used by biologists employing this instrument.

### Results and ratio analyses

Results from microsphere *intraplex *assays where *m *= 4 and *m *= 5 are presented. Several ratios were studied.

For the first ratio, the mean of a set of 6 uncoated microspheres was used as denominator. This mean value was then used to determine a ratio with all the other SMPCS's in each intraplex assay. This is termed an 'external ratio' because it was external to the intraplex set for a single assay.

The second type of ratio was as follows. Since several different intraplex assays were used together (i.e. a multiplexed intraplex), the mean of a different intraplex assay could be explored as a ratio denominator: for example, the ratio of each SRV SMPCS's fluorescent reporter intensity against the *mean *of the SFV SMPCS's fluorescent reporter intensities, and vice versa.

The third ratio is the mean of all values for each intraplex set to their own mean as denominator. Each SMPCS's reading is used as the numerator over the mean of all the values in that set. The ratio of all SMPCS reporters in the intraplex was taken against that mean. This is termed an internal ratio against the self mean.

**Figure 5:**
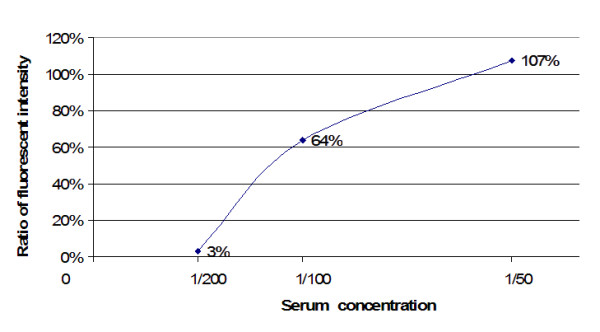
Mean inter-instrument ratio Instrument A/Instrument B. This shows that two different instruments, both under standard service contracts, will not necessarily have the same responses for all concentrations, despite being calibrated using the same standard. This suggests that there is potentially significant variance in the response curves of the parts making up the opto-electronic system. However, the intraplex method eliminated this and other problems.

**Figure 6:**
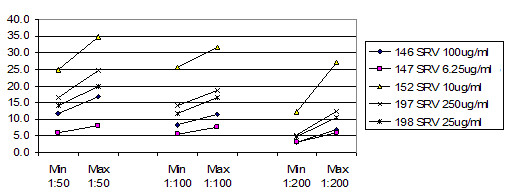
SRV assay external ratios (Y axis) using mean of uncoated microspheres as denominator. (SRV/uncoated mean). (Not used in Table 2.) This is one of two external ratios that were taken. Uncoated microspheres were one of three controls in the experiments, and one of two controls that had multiple SMPCS's. Ranges for three different concentrations of serum are shown, and it is possible to see how ratios cluster closer together as concentration of serum goes down. Compare with figures 7 and 8.

**Figure 7:**
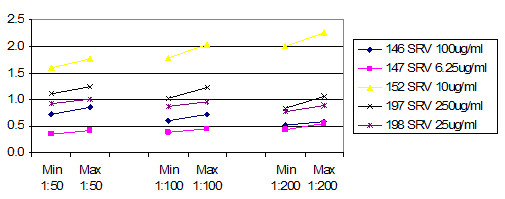
SRV assay external ratios (Y axis) using mean for a different assay set as denominator (SRV/SFV mean). (Corresponds to Table 2 B.)This ratio appears to work better than that shown in figure 6, which is attributed to apparent greater variance in the uncoated sets than is seen in real assays. Ranges for three different concentrations of serum are shown, and it is possible to see how ratios cluster closer together as concentration of serum goes down. Compare with figures 6 and 8.

**Figure 8:**
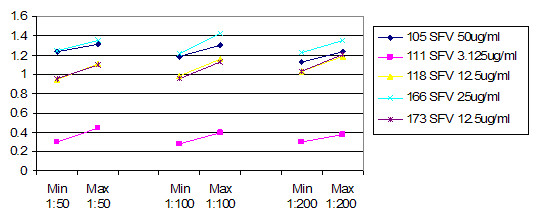
SFV assay internal ratios using internal self mean as denominator (SRV/SRV mean). (Corresponds to Table 2 A.) This ratio conveniently turned out to be the most effective at controlling for all types of variances. Like figures 6 and 7, ranges for three different concentrations of serum are shown, and it is possible to see how ratios cluster closer together as concentration of serum goes down. Compare with figures 6 and 7.

Figure 5 shows the average ratio of raw instrument readings between the two instruments used. Both instruments were calibrated to the same microsphere fluorescence standard, which uses a single point. At 1:50 dilution, the readings were roughly 1:1. This declined to roughly 3:100 for 1:200 dilution for these two instruments. This indicates that the instruments had opto-electronic systems with different response curves. When the concentration decreases, the sets of intraplex ratios cluster closer together (Figure 6, Figure 7 and Figure 8). In addition to stabilizing readings between instruments, this provides the ability to judge the order of magnitude concentration of analyte independently of a concentration standard curve. Figure 6 shows the SRV assay intra-well ratio using the mean of uncoated microspheres as denominator (a type of external ratio). Figures 7 and 8 show SRV microsphere sets using the self mean as denominator (internal ratio).

### Discussion of intraplex ratios

The amount of analysis that could be presented here is considerable. These figures and tables show the essence of what is important for understanding the improvement derived from this new assay technique. The primary work compared results for assay plates with 32 replicate wells where each plate was read on two different instruments.

**Figure 9:**
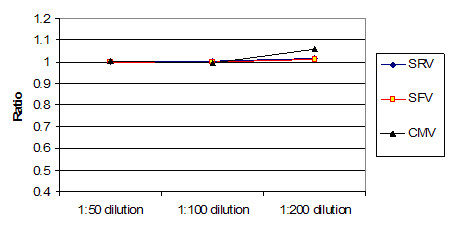
Ratios on internal self mean of set. (Instrument A/Instrument B). As can be seen here, a ratio on the internal self mean gives good correlations between instruments for all three intraplex assays. There is some separation at lower concentrations, which is expected as the signal to noise ratio declines. (Corresponds to Table 2 A.)

**Figure 10:**
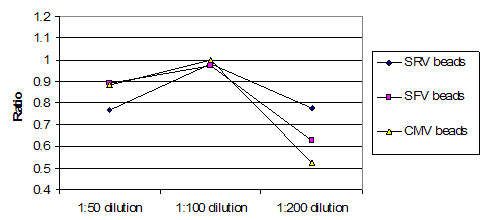
External ratios on uncoated mean. (Instrument A/Instrument B) This figure shows ratios on an external mean, where an external mean is the mean of an assay for a different analyte. This graph demonstrates that, on average, an apparently quite stable external mean is not as good as an internal mean ratio. Comparing Figures 9 and 10, one can see that Figure 9 has ratios that are closer to the desired ratio of 1. (Corresponds to Table 2 B.)

The graphs of Figure 9 and Figure 10 were generated as follows for both instruments:

1. For each well, a ratio between the fluorescent intensity (FI) and several denominators was taken. The denominators were: mean of uncoated control microsphere FI; FI mean of external real assays; FI self mean of the intraplex set; and FI of one arbitrarily selected SMPCS from the intraplex.

2. For each SMPCS, the mean, median, maximum, minimum, and standard deviation were calculated for each 32-well replicate serum titration.

3. Between instruments, the ratios of the mean, median, maximum, minimum and standard deviations were calculated for each serum titration. This was done for each permutation of denominators taken in step 1.

**Figure 11:**
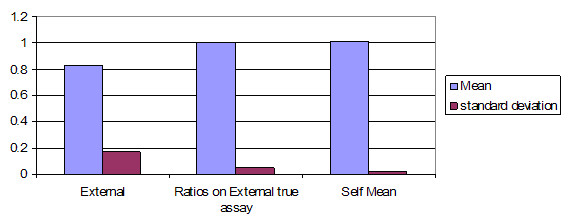
Mean and standard deviation by type of ratio taken. This graph shows the mean inter-instrument comparison of the ratios by type, and their standard deviations by type. What one looks for here is a mean ratio that is closest to one, combined with the smallest standard deviation. In this graph is seen summarized the data seen in different form in figures 6, 7 and 8, respectively for the three items in this graph.

The ratio of means is used for expediency due to the quantity of data in this study. A potentially valid criticism is that this procedure might remove a wide distribution from the system. For this reason, the bar chart of Figure 11 is shown, which compares the mean correlation and shows the standard deviation for each type of correlation. In addition, a difference of means *z *score was calculated for each method and is presented in the next subsection to show that the correlation is valid.

### Difference of means test

The last step of this analysis was to examine the z scores for the intraplex assays with using a difference of means test.

z=X¯1−X¯2s12n1+s12n2
 MathType@MTEF@5@5@+=feaafiart1ev1aaatCvAUfKttLearuWrP9MDH5MBPbIqV92AaeXatLxBI9gBaebbnrfifHhDYfgasaacH8akY=wiFfYdH8Gipec8Eeeu0xXdbba9frFj0=OqFfea0dXdd9vqai=hGuQ8kuc9pgc9s8qqaq=dirpe0xb9q8qiLsFr0=vr0=vr0dc8meaabaqaciaacaGaaeqabaqabeGadaaakeaacqWG6bGEcqGH9aqpdaWcaaqaaiqbdIfayzaaraWccqaIXaqmkiabgkHiTiqbdIfayzaaraWccqaIYaGmaOqaamaakaaabaWaaSaaaeaacqWGZbWCdaqhaaWcbaGaeGymaedabaGaeGOmaidaaaGcbaGaemOBa42aaSbaaSqaaiabigdaXaqabaaaaOGaey4kaSYaaSaaaeaacqWGZbWCdaqhaaWcbaGaeGymaedabaGaeGOmaidaaaGcbaGaemOBa42aaSbaaSqaaiabikdaYaqabaaaaaqabaaaaaaa@420A@

Above, X¯1
 MathType@MTEF@5@5@+=feaafiart1ev1aaatCvAUfKttLearuWrP9MDH5MBPbIqV92AaeXatLxBI9gBaebbnrfifHhDYfgasaacH8akY=wiFfYdH8Gipec8Eeeu0xXdbba9frFj0=OqFfea0dXdd9vqai=hGuQ8kuc9pgc9s8qqaq=dirpe0xb9q8qiLsFr0=vr0=vr0dc8meaabaqaciaacaGaaeqabaqabeGadaaakeaacuWGybawgaqeaSGaeGymaedaaa@2EF8@ and X¯2
 MathType@MTEF@5@5@+=feaafiart1ev1aaatCvAUfKttLearuWrP9MDH5MBPbIqV92AaeXatLxBI9gBaebbnrfifHhDYfgasaacH8akY=wiFfYdH8Gipec8Eeeu0xXdbba9frFj0=OqFfea0dXdd9vqai=hGuQ8kuc9pgc9s8qqaq=dirpe0xb9q8qiLsFr0=vr0=vr0dc8meaabaqaciaacaGaaeqabaqabeGadaaakeaacuWGybawgaqeaSGaeGOmaidaaa@2EFA@ are the mean of the respective reading sets for the two instruments, *n*_1 _and *n*_2 _is the number of readings, *s*_1 _and *s*_2 _are the standard deviations of the samples. For these tests the same set of 32 replicated sample wells was read, once on instrument A followed by repeating the same plate on instrument B, the anticipated results are identical.

The results of this analysis are summarized in Table 2. Examining the table, it is apparent that the best results are for ratios on internal self mean (2A), as these are significantly closer to the optimum ratio of 1.0 that indicates identical readings.

**Table 2:** Comparison of stability between instruments of three methods: A. internal self mean ratio; B. ratios based on an external assay; and C raw instrument data. Internal self mean (A) is the most reliable. Using external ratios, (2C) is a close second, and raw readings, (2C) show the greatest deviation between instruments.

	**Ratios **N = 32 for all.	**Mean z score**	**Median z score**	**Max z**	**Min z**
**A**	**Ratios on internal self mean**				
	Rh. Sera 1:50	1.07	0.84	2.08	0.52
	Rh. Sera 1:100	0.96	0.92	1.21	0.76
	Rh. Sera 1:200	1.86	2.31	3.50	0.14

**B**	**Ratios on real external mean (SRV/SFV mean and SFV/SRV mean)**				
	Rh. Sera 1:50	1.70	1.82	2.50	0.50
	Rh. Sera 1:100	1.44	1.44	3.83	0.27
	Rh. Sera 1:200	16.38	11.97	49.47	4.59

**C**	**Raw inter instrument comparisons**				
	Rh. Sera 1:50	1.19	1.09	1.75	0.61
	Rh. Sera 1:100	8.14	7.91	9.69	6.98
	Rh. Sera 1:200	46.04	43.89	65.40	26.52

## Conclusion

This study indicates that intraplex methodology provides significant benefits to suspended microarray assay precision, and that for an intraplex analysis the ratio to the internal self-mean would be optimal to use, although a developer may choose an external method for some circumstance, or use both internal and external methods together as cross validations. An intraplex should produce reliable results regardless of which specific instrument (appropriate for the assay manufacturer) is used. Intraplex ratios compensated for known assay error modes.

A graph of the internal self-mean clustering will show *n *ratios moving closer together, with a high or low outlier in most instances, since signal response levels will usually vary semi-logarithmically as the analyte concentration is lowered, frequently causing mean of *m *to have an apparent outlier. This clustering provides a measure correlated to concentration of analyte.

To achieve intra-plate standard concentration determination independence, intraplex assays can be run by an assay developer at differing levels of known analyte. Ratios for each analyte assay can then be generated for each intraplex assay batch. These ratios can then be used to provide an independent intra-assay correlation with analyte concentration. To make the assay even more precise, intraplex assays could be used together with the current system of creating a standard curve for each assay plate. Combining such results will allow diagnosis of problems with standard solutions, and provide potentially greater precision.

Intraplexing assays are useful for several purposes. Intraplexing should provide a means of making the serious issue of unpredictable large carryover events[10] visible should they occur, and can compensate for them. An intraplex assay that is carefully calibrated by replication should show a characteristic set of relationships between the components of the assay. Proper analysis of results should enable outlier readings for an SMPCS to be discarded. Thus, an intraplex of 5 to 10 SMPCS's should provide a good degree of accuracy.

Having a value of *n *≥ 5 for the remainder of an *m *× *n *intraplex after culling possible outliers provides useful statistical significance, although some may accept lower values of *n *and some may require higher. The processed data from an individual well, using intraplexing, can have a validity that is currently unavailable, thus avoiding requirements for sample replication in many uses. Validity will be generally based on *t *tests, but with a reasonable confidence. This can allow software vendors to make better judgments for users regarding the statistical significance of a result.

Users of suspended microarray assay systems should take note of this method and apply its results as appropriate to their systems. Much of these results apply to "smart dust", smart microspheres, bar coded microspheres, microrods and others. To confer optimum precision for research, clinical use and other applications on this sector of assay technology, the matters raised here also should be considered for these alternative assay methods. Additionally, users may want to take note of the potential for significant differences between instruments when instruments are calibrated to the same standard.

## Competing interests

The author(s) declare that they have no competing interests.
